# Design and Analysis of Digitally Tunable Transconductance Amplifier (DTTA) Using CNTFETs

**DOI:** 10.1155/2024/2003437

**Published:** 2024-05-23

**Authors:** Shailendra Kumar Tripathi, Sarfraz Hussain, Raj Kumar, Sourabh Sahu

**Affiliations:** ^1^Department of Physics and Material Science, Jaypee University, Anoopshahr, India; ^2^School of ECE, REVA University, Bangalore, India; ^3^Department of ICT, Adani University, Ahmedabad, India; ^4^Department of ECE, Gyan Ganga Institute of Technology and Sciences, Jabalpur, India

## Abstract

Carbon nanotube-FETs (CNTFETs) have become a potential challenger because of their exceptional electrical properties and compatibility with conventional CMOS technology. The design and study of digitally tunable transconductance amplifiers (DTTAs) using CNTFETs are the main topics of this work. By utilizing the special characteristics of CNTFETs, the suggested DTTA design makes transconductance tunable, providing a versatile method of adjusting amplifier settings without requiring modifications to the hardware architecture. This study provides a complete description of the CNTFET modeling techniques utilized for realistic circuit simulations, along with a detailed analysis of the DTTA based on CNTFETs. The circuit is implemented using a 32 nm CNTFET model and verified results with HSPICE.

## 1. Introduction

As semiconductor technology advances, CNTFET-based circuits show the potential to overcome the limits of classical CMOS in developing applications such as high-frequency communication systems, sensor interfaces, and medicinal devices. However, real deployment may need to address concerns about manufacturing scalability, cost-effectiveness, and long-term stability related to CNTFET manufacture and integration [1–3].

Filters having a very low cut-off frequency are required for the processing of physiological signals. The frequency range of commonly utilized physiological signals ranges from sub-Hz to a few KHz. The operational transconductance amplifier (OTA) is a key component in low-frequency analog signal processing. These applications need the development of OTA with extremely low transconductance (*g*_*m*_) [4, 5]. Transconductance amplifiers transform voltage signals into current signals in analog and mixed-signal electronics. The entire efficacy of different applications such as signal processing, communication systems, and sensor interfaces is determined by their performance [6, 7]. The ability to tune amplifier characteristics is crucial in adapting these circuits to varied settings without sacrificing efficiency [8–10].

Transconductance amplifiers based on silicon have intrinsic restrictions, notably in terms of power requirement, operating frequency, and flexibility. As an alternative, integrating CNTFETs provides a convincing solution to these restrictions. CNTFETs' outstanding electron mobility, resilience, and nanoscale dimensions enable circuit designers to push the performance envelope [11, 12].

In this work, we find the feature of carbon nanotube (CNT) for increasing the drain current. This feature is utilized for the digitally tunable transconductance amplifier. Thus, drain current can be controlled by the number of CNTs for a digital control stage. Here, we have discussed the control of drain current depends on the diameter and number of CNTs in a carbon nanotube-FET.

First, in CMOS, the minimum W/L ratio of PMOS to NMOS is 2.5 times. In order to have smooth performance, the W/L ratio of PMOS is usually kept higher than 2.5. NMOS has electrons as majority-charge carriers, and PMOS has holes as majority-charge carriers. Electrons have mobility 2.7 times higher than holes. The main reason behind making PMOS larger is that the rise time and fall time of the gate should be equal and for the resistance of the NMOS and PMOS should be the same. This can be achieved only by sizing the PMOS 3 times to the NMOS sizing. However, in the case of CNFET, pCNFET and nCNFET can have the same width because the mobility in the pCNFET and nCNFET is the same.

**Figure 1:**
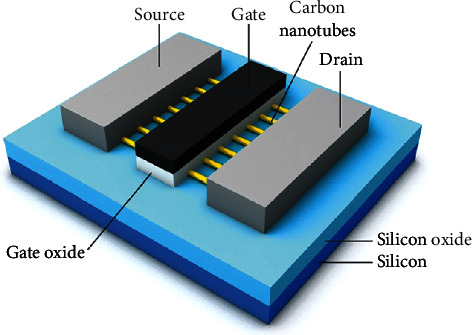
A simplified cross-sectional view of CNFET [16].

This study aims to investigate the design and analysis of digitally tunable transconductance amplifiers (DTTAs) that take advantage of the capabilities of CNTFETs. The major goal is to use CNTFET intrinsic features to improve circuit performance including gain, power efficiency, bandwidth, and linearity [13, 14]. The amplifier's properties may be dynamically modified to accommodate different operating needs by including digital tuning mechanisms, significantly broadening the area of its use [15–18]. A carbon nanotube field effect transistor (CNTFET) has a cross-sectional view as shown in Figure 1.

The paper is structured in the following way. The design aspect of the digitally tunable transconductance amplifier (DTTA) is presented in Section 2.  Section 3 illustrates the simulation results of the presented DTTA while Section 4 concludes the paper.

## 2. Proposed Digitally Tunable Transconductance Amplifier (DTTA)

Two input voltages are used by the operational transconductance amplifier (OTA), which generates an output current proportional to the difference between the two voltages. The output current of OTA is expressed by (1), where *g*_*m*_ is the transconductance of the amplifier, and the biasing current governs its value (*I*_Bias_) applied to the circuit [19–21].

(1) $$\begin{array}{c} I_{\text{out}}=g_{m}*\left(V_{1}-V_{2}\right). \end{array}$$

Further, the design parameters of CNTFET are given in Table 1.

The design aspect and parameters are kept in consideration for the CNFETs. The CNFETs have wide flexibility in terms of the width of the CNFET-based transistor (W), number of CNTs in the channel (N), and inter-CNT spacing (S).

In this work, CNTs are utilized which have very high drive currents, less scattering, and near ballistic transport of charge carriers. The feature of the carbon nanotube is explored by which the drain current in a transistor is controlled by CNTs in the channel. This property can be utilized to design a DTTA. It enhances the operability and flexibility of the transconductance amplifier. The proposed method uses multiple CNTs to increase the drain current of the transistor.

The proposed circuit is novel as the following design parameters of CNFET are considered: the CNT's diameter (DCNT), the width of the CNFET-based transistor (W), the number of CNTs in the channel (N), and the inter-CNT spacing (S). The width of CNFETs depends on the number of CNTs used in each stage and is calculated by the following equation:

(2) $$\begin{array}{c} W=\left(N-1\right)*S+D_{\text{CNT}}, \end{array}$$

where *D*_CNT_ is the diameter of CNT, S is interspacing between CNTs, and N is the number of CNTs in CNFET. In the given circuit, the transistor stages (*M*_1_ − *M*_14_) and (*M*_27_ − *M*_30_) have four CNTs. Further, to double the current in the next stage (*M*_15_ − *M*_18_) and (*M*_31_ − *M*_34_), the number of CNTs should be doubled, i.e., eight. Similarly, stages (*M*_19_ − *M*_22_) and (*M*_35_ − *M*_38_) contain sixteen CNTs.

**Figure 2:**
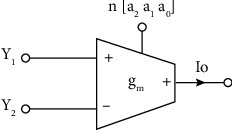
Schematic representation of DTTA.

The digitally tunable approach is now being used for the transconductance amplifier. Many digitally adjustable approaches have been described in the literature [22–25]. The digital control approach also increases the circuit's reconfigurability. A digitally controlled voltage gain amplifier (VGA) with a CMOS digitally programmable current conveyor was presented in 2008 [22]. A current-controlled conveyor (CCC-II) with digital control via a current division network (CDN) has also been proposed to achieve a multiphase oscillator [23]. A simplified block diagram of the proposed DTTA is shown in Figure 2. The voltage is applied through terminal *Y*_1_ and *Y*_2_, and the output is taken from *I*_0_ terminal. Additionally, “*n*” represents the control word (*a*_2_; *a*_1_; *a*_0_) which is used to tune the transconductance.

Furthermore, the authors [24] describe a CMOS-based digitally programmable current conveyor-II that employs four bits to regulate the Z+ terminal's current. Next, to realize the digitally programmable current follower (DP-CF), the current division cell (CDC) is used in the approaches as described in [25].

While, in carbon nanotube-FETs, the drain current increases by increasing the number or diameter of CNTs, in MOSFETs, the drain current (*I*_*D*_) is enhanced by raising the transistor width [15, 24]. This implies that by doubling the number of tubes in a carbon nanotube-FET, we can double the current.

### 2.1. Circuit Description

**Figure 3:**
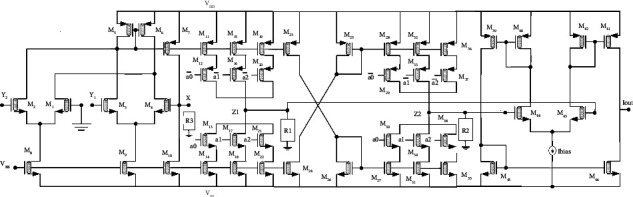
CNFET-based implementation of the proposed digitally tunable transconductance amplifier (DTTA).

Figure 3 illustrates the suggested digitally tunable transconductance amplifier (DTTA). Transistors *M*_1_ and *M*_2_ and *M*_3_ and *M*_4_ constitute two differential amplifiers driven by transistors M5 and M6, which function as a current mirror. Furthermore, transistors *M*_7_ and *M*_10_ provide the necessary feedback action to ensure that the voltage *V*_*X*_ is independent of the current pulled from terminal X. Additionally, the total of *M*_1_ and *M*_4_ drain currents equals *M*_2_ and *M*_3_ drain currents. Moreover, the drain currents of transistors *M*_8_ and *M*_9_ would be equivalent as they are biased with comparable gate voltages (and their source voltages are also equal). This would get the following for matching *M*_8_ and *M*_9_:

(3) $$\begin{array}{c} V_{X}=\left(V_{Y1}-V_{Y2}\right). \end{array}$$

The current at terminal *X*(*I*_*X*_) is transferred as *n∗I*_*X*_ to the *Z*_1_ terminal through transistors *M*_7_, *M*_10_ − *M*_22_. The current is delivered −*n∗I*_*X*_ to the *Z*_2_ terminal by employing an additional current mirror stage (*M*_23_ − *M*_38_).

The voltages *V*_*Z*1_ and *V*_*Z*2_ develop at node *Z*_1_ and *Z*_2_, which are further applied to gates of *M*_44_ and *M*_45_ transistors. The transistors *M*_39_ − *M*_46_ form a structure to generate the output current (*I*_0_) which is proportional to the differential inputs, *V*_*Z*1_ and *V*_*Z*2_.

In the given circuit, transistor stages (*M*_1_ − *M*_14_) and (*M*_27_ − *M*_30_) have four CNTs. Further, to double the current in the next stage (*M*_15_ − *M*_18_) and (*M*_31_ − *M*_34_), number of CNTs should be doubled, i.e., eight. Similarly, stages (*M*_19_ − *M*_22_) and (*M*_35_ − *M*_38_) contain sixteen CNTs. The equation for the current can be represented as follows:

(4) $$\begin{array}{c} I_{Z1}=n*I_{X};I_{Z2}=-n*I_{X}. \end{array}$$

Here, “*n*” represents the digitally tunable word. For a 3 bit digital tunable word, its value ranges from 0 to 7.  Table 2 illustrates the dependency of current gain on the digital tunable word (*n*). Further, in the next stage, transistors (*M*_39_ − *M*_42_) have four CNTs and transistors (*M*_43_ − *M*_46_) have one CNT. For the suggested DTTA, the supply voltage and bias voltage (*V*_*BB*_) have been maintained at 0.7 V and −0.36V, respectively. The detailed dimensions of CNFETs are given in Table 3.

For the currents *I*_*X*_ and *I*_*Z*_, the circuit analysis of Figure 3 yields the relations (6) and (7). It is also possible to acquire the voltages of the *Z*_1_ and *Z*_2_ nodes using (8). The expression for *V*_*Z*1_ and *V*_*Z*2_ may be expressed as (9) after applying the values of *I*_*Z*1_ and *I*_*Z*2_.

(5) $$\begin{array}{c} I_{X}=\frac{V_{X}}{R_{3}}=\frac{\left(V_{Y1}-V_{Y2}\right)}{R_{3}}, \end{array}$$

(6) $$\begin{array}{c} I_{Z1}=n*I_{X};I_{Z2}=-n*I_{X}, \end{array}$$

(7) $$\begin{array}{c} I_{Z1}=n*\frac{\left(V_{Y1}-V_{Y2}\right)}{R_{3}};I_{Z2}=-n*\frac{\left(V_{Y1}-V_{Y2}\right)}{R_{3}}, \end{array}$$

(8) $$\begin{array}{c} V_{Z1}=I_{Z1}*R_{1};V_{Z2}=I_{Z2}*R_{2}, \end{array}$$

(9) $$\begin{array}{c} V_{Z1}=n*\left(V_{Y1}-V_{Y2}\right)*\frac{R_{1}}{R_{3}};V_{Z2}=-n*\left(V_{Y1}-V_{Y2}\right)*\frac{R_{2}}{R_{3}}. \end{array}$$

Considering the characteristics of the OTA, the output current expression may be expressed as follows: (10). If *R*_1_=*R*_2_=*R*, *R*_3_=2*R* and putting the value of *V*_*Z*1_ and *V*_*Z*2_, we find (11) and (12).

(10) $$\begin{array}{c} I_{\text{out}}=g_{m}*\left(V_{Z1}-V_{Z2}\right), \end{array}$$

(11) $$\begin{array}{c} I_{\text{out}}=n*g_{m}*\left[\frac{\left(V_{Y1}-V_{Y2}\right)}{2}-\left(-\frac{\left(V_{Y1}-V_{Y2}\right)}{2}\right)\right], \end{array}$$

(12) $$\begin{array}{c} I_{\text{out}}=\left(n*g_{m}\right)*\left(V_{Y1}-V_{Y2}\right). \end{array}$$

By comparing equations (1) and (12), it is seen that *g*_*m*_ is controlled by the tunable word “n”.

The output current and transconductance are also related. Equations (11) and (12) give the relation between the output current and transconductance.

## 3. Simulation Results

**Figure 4:**
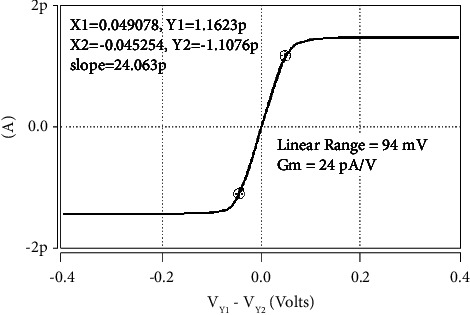
Linear range of the proposed DTTA.

**Figure 5:**
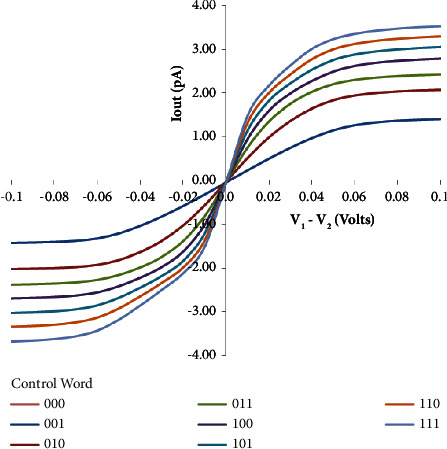
Representation of linear ranges vs. tunable bits for the DTTA.

Additionally, Figure 4 depicts the linear range of the digitally tunable transconductance amplifier (DTTA) of Figure 3. Here, the resistors *R*_1_=*R*_2_=1*K* and *R*_3_=2*K* take the value of the digitally tunable word (*n*) as one. Transconductance has a linear range of 94 mV and a simulated value of 24 pA/V. DTTA has two maximum and lowest values, 24 pA/V and 140 pA/V, respectively. The DTTA's linear range varies from 22 mV to 94 mV. The fluctuation in the DTTA's linear range with the digitally adjustable word is shown in Figure 5.

With a change in the tunable word (*n*), Table 4 displays the linear ranges and simulated transconductance values of the digitally tunable transconductance amplifier (DTTA). This indicates that there is flexibility in selecting alternative transconductance values using the proposed circuit.

The digital inputs are applied through the digital word (*a*_2_; *a*_1_; *a*_0_) which are the gates of transistors. The current at terminal *X*(*I*_*X*_) is transferred as *n∗I*_*X*_ to the *Z*_1_ terminal through transistors *M*_7_, *M*_10_ − *M*_22_. The current is delivered −*n∗I*_*X*_ to the *Z*_2_ terminal by employing an additional current mirror stage (*M*_23_ − *M*_38_). The voltages *V*_*Z*1_ and *V*_*Z*2_ develop at nodes *Z*_1_ and *Z*_2_, which are further applied to the gates of *M*_44_ and *M*_45_ transistors. The transistors *M*_39_ − *M*_46_ form a structure to generate the output current (*I*_0_) which is proportional to the differential inputs, *V*_*Z*1_ and *V*_*Z*2_ as discussed in Section 2.1.

## 4. Conclusion

This study contributes to the integration of sophisticated nanoscale devices into analog circuit design by providing a digitally tunable transconductance amplifier (DTTA) based on CNTFETs. The circuit uses 0.7V of power supply. The range of transconductance achieved by the proposed DTTA is 24 pA/V-140 pA/V through the tunable word (000–111) with a linear range of 22 mV–94 mV. The reported results highlight the potential of this technique to change circuit design approaches, providing new levels of flexibility and performance for future electronic systems. These design approaches may help the progress of current electronic systems by improving circuit performance and providing flexibility, opening the way for more efficient and adaptable integrated circuits in the future. Continued research and development in this area will be critical in achieving the full potential of low-power CNTFET-based circuits for a variety of applications.

## Figures and Tables

**Table 1:** CNTFET parameters for the proposed DTTA.

Parameters	Value
Oxide thickness (*T*_ox_)	4 nm
Dielectric constant (*K*_ox_)	16
Power supply	±0.7V
Chirality of the tube (*n*, *m*)	19, 0
Physical channel length (*L*_ch_)	32 nm
Pitch (*S*)	20 nm
Diameter of CNT (*D*_CNT_)	1.5 nm

**Table 2:** Tunable bits for proposed DTTA.

*a*_2_ (*n*)	*a*_1_ (*n*)	*a*_0_ (*n*)	Current gain (*I*_*Z*_/*I*_*X*_)
0	0	0	0
0	0	1	1
0	1	0	2
0	1	1	3
1	0	0	4
1	0	1	5
1	1	0	6
1	1	1	7

**Table 3:** Dimensions of CNFETs.

Transistor stages	Width (W) (nm)	Length (L) (nm)
(*M*_1_ − *M*_14_) and (*M*_27_ − *M*_30_)	61.5	32
(*M*_15_ − *M*_18_) and (*M*_31_ − *M*_34_)	141.5	32
(*M*_19_ − *M*_22_) and (*M*_35_ − *M*_38_)	301.5	32

**Table 4:** Linear range and transconductance (*g*_*m*_) Values of DTTA.

S.N.	Tunable word (*n*)	*g*_*m*_ (pA/V)	Linear range (mV)
1	000	0	0
2	001	24	94
3	010	42	73
4	011	60	53
5	100	75	45
6	101	90	40
7	110	120	25
8	111	140	22

## Data Availability

We believe that ensuring that the data underlying the findings of a paper are publicly available wherever possible—as open as possible and as closed as necessary—will help ensure that the work described in an article can potentially be replicated. We therefore firmly support and endorse the FAIR Guiding Principles for scientific data management and stewardship—that are Findability, Accessibility, Interoperability, and Reusability. There are many benefits to sharing data—it increases not only the utility and reliability of your work but also its impact and visibility and your profile and credibility as a researcher.
